# Exploring bi-carbazole-linked triazoles as inhibitors of prolyl endo peptidase via integrated in vitro and in silico study

**DOI:** 10.1038/s41598-024-58428-6

**Published:** 2024-04-01

**Authors:** Saeed Ullah, Farheen Mansoor, Salman Ali Khan, Uzma Jabeen, Amany I. Almars, Hailah M. Almohaimeed, Ahmed M. Basri, Fahad M. Alshabrmi

**Affiliations:** 1 Dr. Panjwani Center for Molecular Medicine and Drug Research, International Center for Chemical and Biological Sciences, University of Karachi, Karachi, 75270 Pakistan; 2 Tunneling Group, Biotechnology Centre, Doctoral School, Silesian University of Technology, Akademicka 2, 44-100 Gliwice, Poland; 3 Department of Biochemistry, Federal Urdu University of Karachi, Gulshan-e-Iqbal, Karachi, 75300 Pakistan; 4 Department of Medial Laboratory Sciences, Faculty of Applied Medical Science, King Abdulaziz University, 21589 Jeddah, Saudi Arabia; 5 Department of Basic Science, College of Medicine, Princess Nourah Bint Abdulrahman University, P.O. Box 84428, 11671 Riyadh, Saudi Arabia; 6 Department of Medical Laboratories, College of Applied Medical Sciences, Qassim University, 51452 Buraydah, Saudi Arabia

**Keywords:** Bi-carbazole-linked triazoles, In vitro, Molecular docking simulation, Prolyl endo peptidase inhibitory activity, Kinetic studies, Chemical biology, Computational biology and bioinformatics

## Abstract

A serine protease called prolyl endopeptidase (PEP) hydrolyses the peptide bonds on the carboxy side of the proline ring. The excessive PEP expression in brain results in neurodegenerative illnesses like dementia, Alzheimer’s disease, and Parkinson's disease. Results of the prior studies on antioxidant activity, and the non-cytotoxic effect of bi-carbazole-linked triazoles, encouraged us to extend our studies towards its anti-diabetic potential. Hence, for this purpose all compounds **1**–**9** were evaluated to reveal their anti-prolyl endo peptidase activity. Fortunately, seven compounds resulted into significant inhibitory capability ranging from 26 to 63 µM. Among them six compounds **4**–**9** exhibited more potent inhibitory activity with IC_50_ values 46.10 ± 1.16, 42.30 ± 1.18, 37.14 ± 1.21, 26.29 ± 0.76, 28.31 ± 0.64 and 31.11 ± 0.84 µM respectively, while compound **3** was the least active compound in the series with IC_50_ value 63.10 ± 1.58 µM comparing with standard PEP inhibitor bacitracin (IC_50_ = 125 ± 1.50 µM). Moreover, mechanistic study was performed for the most active compounds **7** and **8** with *K*_*i*_ values 24.10 ± 0.0076 and 23.67 ± 0.0084 µM respectively. Further, the in silico studies suggested that the compounds exhibited potential interactions and significant molecular conformations, thereby elucidating the structural basis for their inhibitory effects.

## Introduction

Prolyl oligopeptidase, also known as prolyl endopeptidase (PEP, EC 3.4.21.26) or post-proline cleaving enzyme, catalyzes the hydrolysis of short peptides with less than 30 amino acid residues^1^. PEP is a member of the distinctive peptidase S9A family of serine proteases^2^. Dipeptidyl peptidase IV (DPP IV, EC 3.4.14.5), acylamino acyl-peptidase (EC 3.4.19.1), and oligopeptidase B are further representative members of this family (EC 3.4.21.83)^3^. Although none of these enzymes are proline-specific, their sequence homology around the active triad allows them to be classified as members of the same evolutionary family. Contrary to the traditional serine protease families, such as subtilisin and trypsin, this family only degrades small peptides while leaving larger proteins intact^4,5^. PEP expression or activity levels differ between tissues or regions of tissues, according to several reports. PEP activity was discovered in all of the more than 20 human tissues that had been examined, although the cerebral cortex of the human brain had the highest activity^6^. The brain of the rat, which is frequently utilized as a model animal in several performance tests, exhibited the highest PEP activity, and the activity was more evenly distributed across the brain^6^.

PEP's true physiological function is not entirely understood, however various pathologic events have been linked to overexpression or under expression of PEP. Prior research revealed that PEP activity is linked to important physiological processes such cell differentiation and division, memory and learning, and signal transmission^7^. Neuropeptides and peptide hormones such oxytocin, bradykinin, angiotensin II, arginine-vasopressin, and beta-endorphin have been demonstrated to be inactivated by extracellular PEP. PEP has also been linked to the degradation of IP3, a crucial component of the neuropeptide signaling transduction pathway (inositol-1,4,5-P3). By attaching to their receptors on the endoplasmic reticulum membrane, neuropeptides boost IP3 levels and cause the release of Ca^2^^+^, which is thought to be essential for memory and learning^8^. PEP inhibitors have also been demonstrated to have cognitive-improving and neuroprotective benefits in a variety of animal models^9^.

An efficient therapeutic strategy for treating neurodegenerative illnesses would be any medication that could control the amounts of neuroactive peptides. Recent studies demonstrated the involvement of prolyl endopeptidase in a number of neurodegenerative illnesses, including Alzheimer's (AD), Parkinson's (PD), and depression^10,11^. PEP inhibiting drugs can speed up autophagy, which minimizes protein accumulation. Autophagy is a cell's internal recycling system that generally removes protein clumps^12^. Potential therapeutic strategies for the management of several neurodegenerative disorders include PEP inhibition. Although three PEP inhibitors, S 17092, JTP-4819, and Z-321, began phase I and phase II clinical studies in the late 1990s, these trials were terminated soon after because there are currently no inhibitors on the market^13,14^.

Parkinson's disease (PD) and other synucleinopathies are facilitated by the protein α-synuclein^15^. It has been demonstrated that PEP may speed up the aggregation of α-synuclein. PEP inhibitors can prevent this action, which lowers α-synuclein aggregation both in vitro and in vivo^16,17^. Thus, inhibiting PEP-mediated seeding and increasing autophagic flow are two ways that PEP inhibition affects α-synuclein aggregation. As a result, inhibiting PEP activity represents a novel possibility for altering α-synuclein in the treatment of PD and other synucleinopathies^11^. With an estimated prevalence of 4% in those over the age of 60, AD is the most prevalent neurodegenerative disease and a leading cause of dementia in the elderly. In the AD brain, neuronal cells produce PEP most frequently and have neurodegenerative morphology^11^. Intriguingly, the avoidance of beta-amyloid deposits in mouse and the neuroblastoma cell line has been reported for two PEP inhibitors, Y-29794 and JTP-4819^3^. One of the main causes of AD is the formation of beta-amyloid plaques^18^. Clinical studies for ß-amyloid have not been very successful since, in AD, this protein collects outside of cells whereas a different protein termed Tau aggregates inside of cells. However, it has sparked attention in Tau, which is currently thought to be the primary factor contributing to neuronal death in AD^19^. The possibility that this enzyme may be engaged in pharmacologically significant activities led chemists to focus on developing efficient and targeted PEP inhibitors. Only a very small number of documented PEP inhibitors are natural; the majority are synthetic^20^.

**Figure 1:**
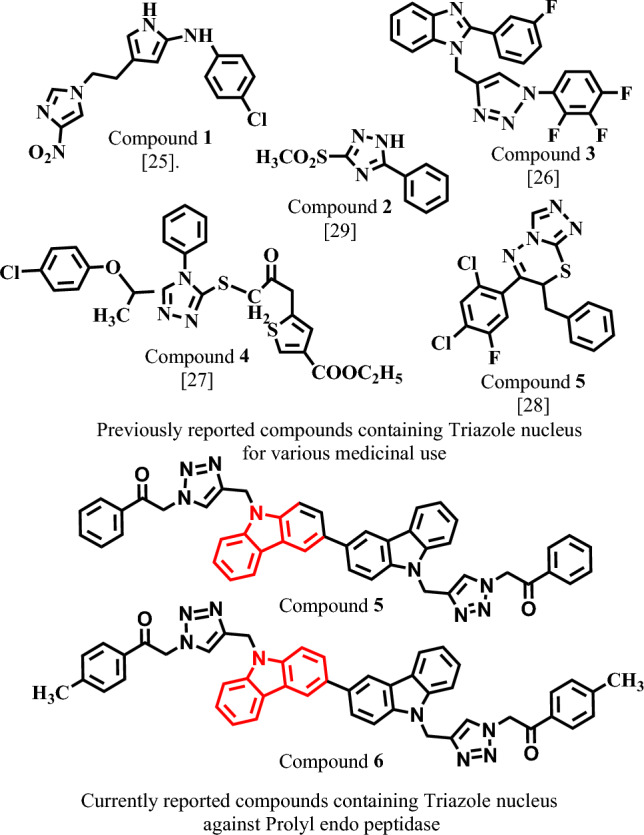
The structures of previously reported and currently reported triazole scaffold as prolyl endopeptidase ligands.

The already reported medicinal importance of triazole comprising compounds encouraged us to evaluate these compounds for their in vitro anti PEP activity (Fig. 1)^21–25^.

Triazoles are very well-known compounds due to diversity in their chemical structure and pharmacological activities. Some of these activities are mentioned for instance, its therapeutic role in human breast cancer and in HIV produced disorders regarding to immune system as well possess anti-bacterial properties^26^. Also reported for anti-tyrosinase^26^, anti-amoebic^27^, anti-inflammatory^28^, anti-plasmodial^29^ and anti-tumor activities^30^ and these compounds are also reported for their anti-oxidant activity^31^. Moreover, there are noteworthy uses for the Triazole Forming Click Chemistry in almost all developing domains. For instance, the structural diversity, biocompatibility, bioavailability, hydrophilicity, and superior ADME properties with minimal toxicity of carbohydrates, an essential component of living cells^32^. Therefore, we decided to explore the medicinal importance of bi-carbazole-linked triazoles. Thus, we proceeded total seven compounds for their anti-prolyl endo peptidase activity to insight into their therapeutic role in neurodegenerative disorders. Moreover, one of the most significant and well-known heterocycles, the triazole nucleus is a typical and essential component of a number of natural products and therapeutic agents used for treatments. A wide variety of drug categories, including those for antimicrobial, anti-inflammatory, analgesic, antiepileptic, antiviral, antineoplastic, antihypertensive, antimalarial, local an aesthetic, antianxiety, antidepressant, antihistaminic, antioxidant, antitubercular, and anti-Parkinson's, contain the triazole nucleus as a key structural component^33^.

## Results and discussion

### Chemistry

**Scheme 1:**
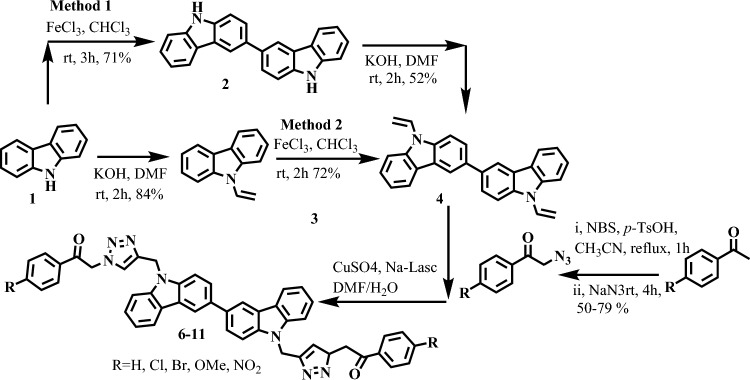
Synthesis of metal catalyzed oxidative coupling reaction for the synthesis of bi-carbazole acetylene (**4**).

Starting with a readily available commercial carbazole (1), a small library of bi-carbazole-linked triazoles (6–11) was created, as shown in Scheme 1. According to our earlier findings^34^, carbazole acetylene (3) was initially made by nucleophilically substituting propargyl bromide into position one. The synthesis of bi-carbazole acetylene (4) was carried out using a metal-catalysed oxidative coupling method. For the synthesis of bi-carbazole acetylene, a variety of metal catalysts were examined (4). Among the other metal catalysts, only FeCl_3_ anhydrous was regarded as an effective catalyst since it produced an oxidatively homo coupled product (4) while the other metals did not catalyse the reaction (Table 1).

**Table 1:** In vitro prolyl endo peptidase inhibitory activity of Bis carbazole 1–9.

Compound	R_1_	R_2_	(IC_50_ ± µM)
1		H	N/A
2		H	N/A
3	H		63.10 ± 1.58
4			46.10 ± 1.16
5			42.30 ± 1.18
6			37.14 ± 1.21
7			26.29 ± 0.76
8			28.31 ± 0.64
9			31.11 ± 0.84
Standard	Bacitracin		125 ± 1.50

Later, two different methods were used to produce bi-carbazole acetylene (4). The first method involved alkylating carbazole (1) with propargyl bromide to produce bi-carbazole (2) (with a 71% yield), which was then converted into 4 in a second stage with a 52% yield (Method 1). Acetylene 3 is directly treated to iron-catalyzed oxidative coupling in a different method that yields bi-carbazole acetylene (4) in a 72% yield (Method 2). Given that the second way produced noticeably superior yields, it is regarded as a workable method (Scheme 1). Bi-carbazole acetylene (4) was then produced under various conditions using the second method (Scheme 1). As shown in scheme, an increase in percent yield was seen when iron chloride (FeCl_3_) anhydrous equivalences were changed from 2.0, 4.5, and 5.0 to 5.5, and a decrease in percent yield was seen because of the degradation of the product when reaction time was increased from 1 to 2 h. The structures of all these compounds were confirmed by using spectroscopic techniques including, Fourier-transform infrared spectroscopy (FTIR) and nuclear magnetic resonance (NMR) and mass-spectrometry (MS) methods^31^.

### Biology

#### In vitro evaluation against prolyl endo peptidase

In the current study nine synthetic derivatives of bi-carbazole linked triazole were subjected for their in-vitro inhibitory study by targeting prolyl endo peptidase to reveal their therapeutic potential against neurodegenerative disorders. Compounds **1**, and **2** displayed below 50% inhibition and were considered non active, while other compounds **3**–**9** resulted into above 50% inhibition and were found to be active against PEP. Interestingly all compounds displayed more activity with IC_50_ values in the range of 26–63 µM (Table 1), while comparing with the standard bacitracin (IC_50_ = 125 ± 1.50 µM). On the other hand, carbazole dimers were found to be several times potent such as compound **3** with addition of carbazole at R^2^ (bi-carbazole) displayed an overwhelming inhibitory capability with IC_50_ value 63.10 ± 1.58 µM, as compared to the standard bacitracin. In compound **4** the addition of propargyl groups at both R^1^ and R^2^ slightly improved the inhibitory potential against PEP with IC_50_ value 46.10 ± 1.16 µM, while comparing with compound **3**. Compound **5** with the addition of triazole of bi-carbazole at R^2^ and a triazole at R^1^ further increased the PEP inhibitory potential with IC_50_ value 42.30 ± 1.18 µM.

Compounds **6–9** have different R^1^ and R^2^ *para* substitutions and these substituents showed no inverse effect on the PEP inhibitory potential, and all compounds exhibited almost similar inhibitory capability against prolyl endo peptidase. For instance, compound **6**, with methyl substitution at both R^1^ and R^2^ para positions showed potent inhibitory activity (IC_50_ = 37.14 ± 1.21 µM), as compared to compounds **3**–**5**. Compound **7** with chloro substitutions at R^1^ and R^2^ *para* positions was found the most potent (IC_50_ = 26.29 ± 0.76 µM) in the series. In compound **8** bromo groups substitution at R^1^ and R^2^ *para* positions showed a very slight decrease in the α-glucosidase inhibitory potential (IC_50_ = 28.31 ± 0.64 µM), as compared to compound **7**. Compound **9**, with methoxy groups substitutions at R^1^ and R^2^ *para* positions showed almost similar inhibitory potential (IC_50_ = 31.11 ± 0.84 µM). Structure activity relationship revealed that different R^1^ and R^2^ substitution displayed important role in the variation of the prolyl endo peptidase inhibitory capability.
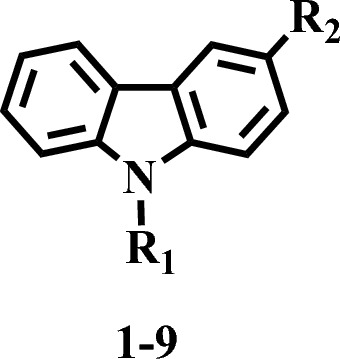


### Kinetic studies

**Figure 2:**
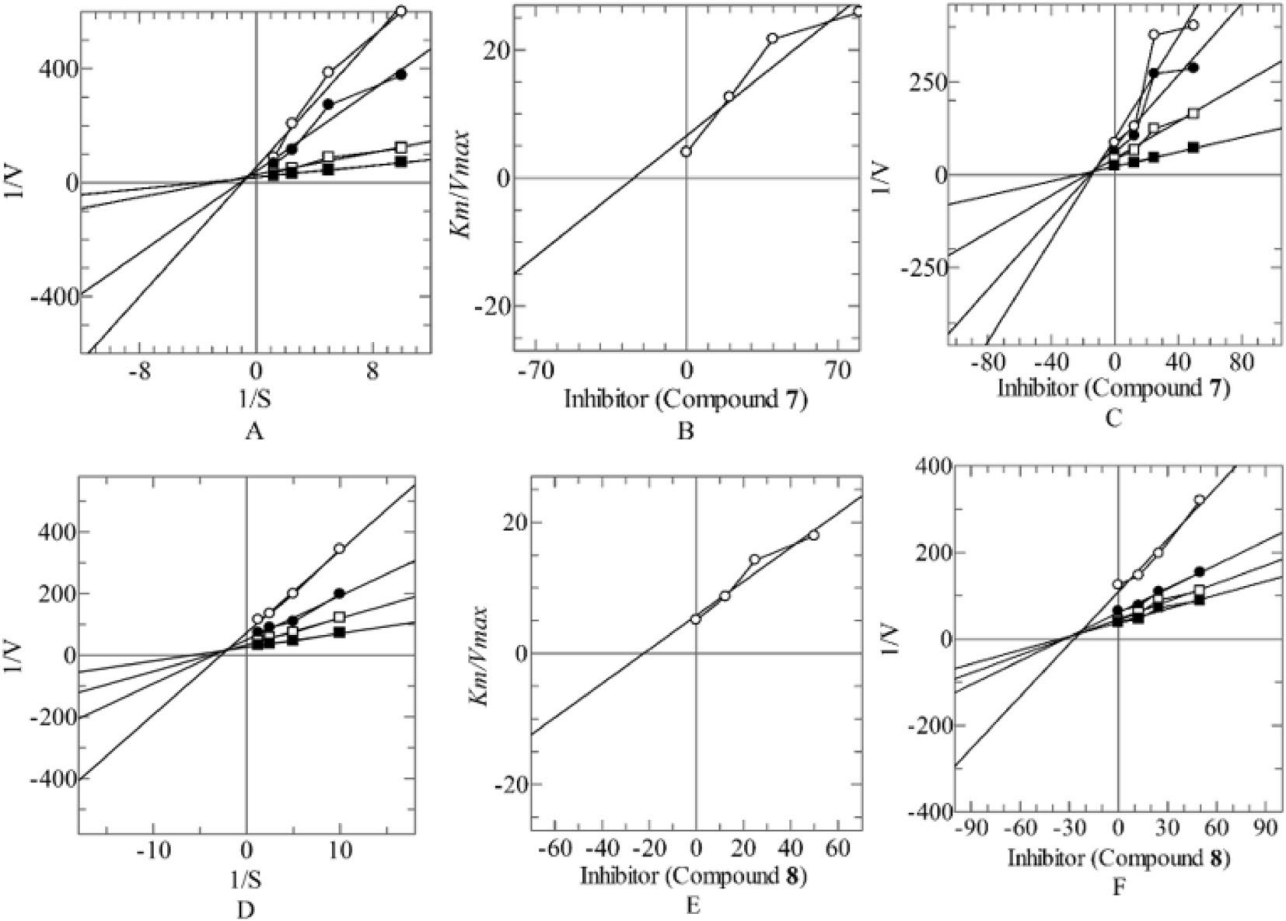
Mode of inhibition of prolyl endo peptidase of compounds **7**, and** 8** (**A**, **D**) Line weaver-Burk plot of reciprocal of rate of reaction (velocities) versus reciprocal of substrate (Z-Gly-Pro-4-nitroanilide) in the absence of compounds** 7** and** 8** (■), and in the presence of compounds **7**, **8** at 50.00 µM (○), 25.00 µM (●), and 12.50 µM (□) respectively. Secondary replot of Line weaver-Burk plot between the slopes of each line on-Line weaver-Burk plot versus different concentrations of compounds **7, 8** (**B**, **E**). Dixon plot of reciprocal of rate of reaction (velocities) versus different concentrations of compounds **7, 8** (**C**, **F**).

To insight into their mechanism of action highly potent compounds **7** and **8** were selected to proceed for kinetic studies. Both compounds resulted into a mixed pattern of inhibition, with *K*_*i*_ values 24.10 ± 0.0076 and 23.67 ± 0.0084 µM. The dose curve of compounds 7 and 8 is provided in supplementary Figure S1-S2. This demonstrate that these new PEP inhibitors mixed type of inhibitors. In such type of inhibition, the inhibitor either binds with the active site residue or other than active site residues. In this case the *Km* hallways increases while *Vmax* of the enzyme decreases (Fig. 2).

### Results of the cytotoxicity study

To identify the adverse effect of compounds **3**–**9**, were subjected for their cytotoxicity study against human fibroblast BJ cell line to reveal its safe use for medicinal importance (Supplementary Figure S3-S5). In the current study interestingly we identified all compounds with safe range, as our findings revealed that all of them displayed below 25% adverse effect on BJ cell line. Hence, the 70% cells viability is an impressive level of non-cytotoxic effect of these compounds (Table 2). Keeping in mind all these findings, these new identified inhibitors can be used as a drug candidates for the therapeutic approach of neurodgenerative disorders.

**Table 2:** In vitro cytotoxicity results of compounds **3**–**9.**

Compounds	% Inhibition of cell viability (60 µM)			% Inhibition ± SEM (µM)	Cytotoxicity effect
3	26.34	22.74	23.95	24.35 ± 1.10	Non-toxic
4	22.75	21.55	26.34	23.55 ± 1.44	Non-toxic
5	25.14	22.74	20.35	22.75 ± 1.38	Non-toxic
6	21.55	20.35	23.95	21.95 ± 1.05	Non-toxic
7	20.35	26.34	25.14	23.95 ± 1.83	Non-toxic
8	19.52	25.14	25.14	23.27 ± 1.87	Non-toxic
9	16.75	21.55	19.16	19.16 ± 1.38	Non-toxic

### Molecular docking study

The PEP enzyme comprises two domains: the catalytic domain and the seven-bladed β-propeller domain. The active site or substrate binding site is situated at the interface between these two domains and is defined by a catalytic triad composed of Ser554, Asp641 and His680^35^. The substrate binding site consisting of three subsites namely, S1, S2 and S3. The S1 subsite is shaped by the side chains of Phe476, Asn555, Val580, Trp595, Tyr599, and Val644. Similarly, the S2 subsite is shaped by the guanidinium side-chain of Arg643 while S3 subsite is shaped by the side chains of Phe173, Met235, Cys255, Ile591, and Ala594^36^. The S1 an S3 subsites were largely hydrophobic in nature while S2 is comparatively polar^35^.

**Figure 3:**
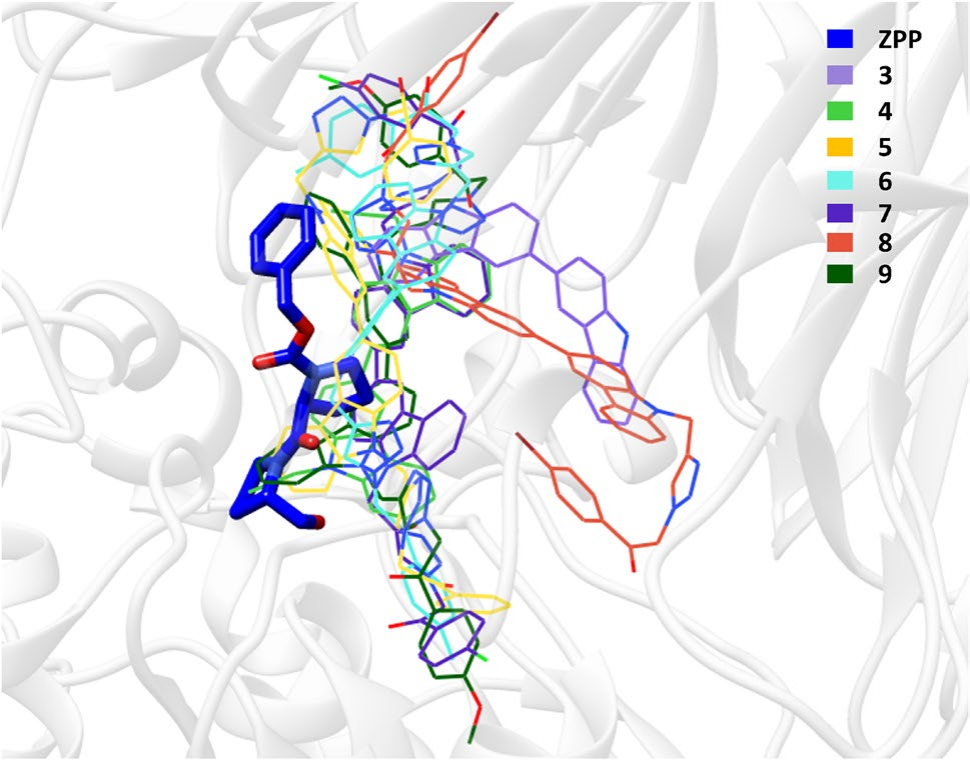
Binding modes of substrate (ZPP) and compounds 3–9 in the PEP binding site. The substrate is displayed in stick representation, while the other compounds are depicted in wire representation for clarity. All compounds are observed to bind in close proximity to the substrate binding site, indicating potential inhibitory activity.

Since the kinetic study of compounds **7** and **8** showed the mixed type of inhibition, we performed the docking with substrate bound (targeted docking) and substrate free (blind docking) form of PEP enzyme to explore the potential binding site. The binding energies from both docking runs were evaluated to determine the binding mechanism of each compound. It was observed that the top-ranked poses of all the compounds bind near or within the substrate binding site (Fig. 3).

**Figure 4:**
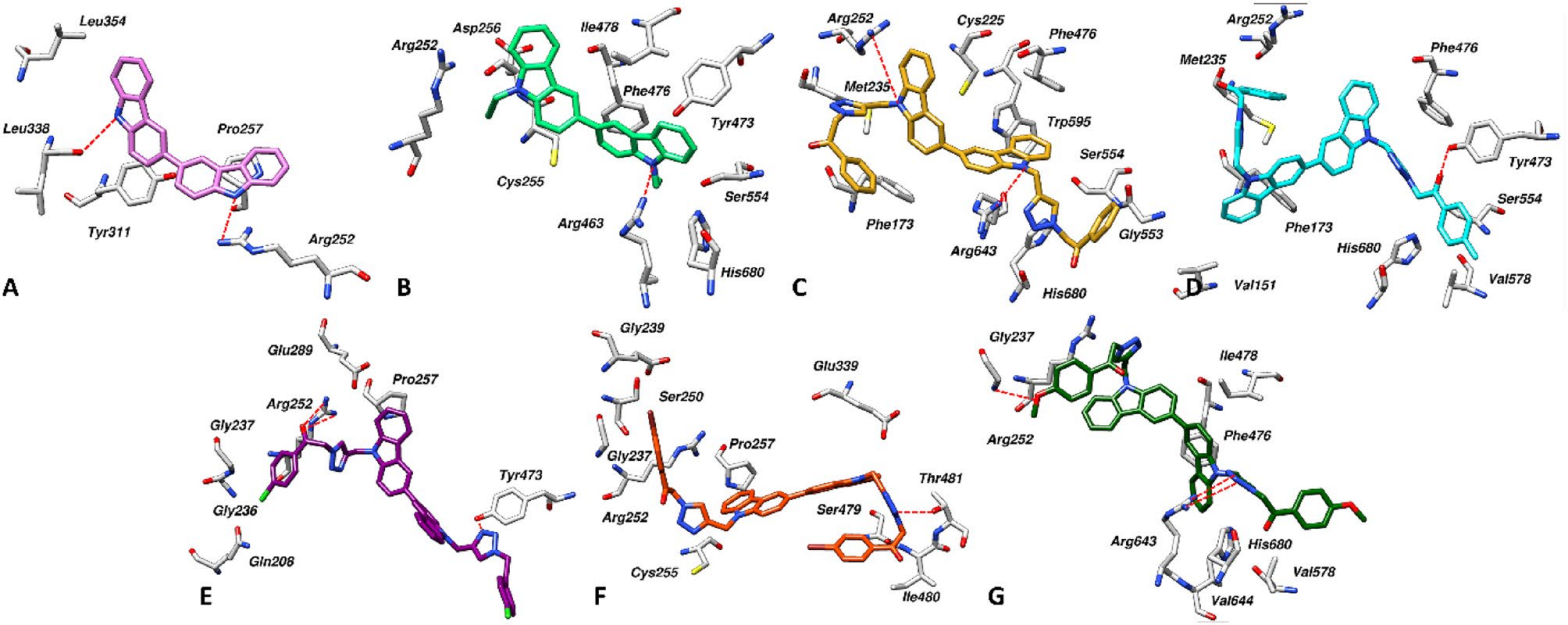
(**A**)–(**G**) Docking interactions of compounds **3–9** with the PEP enzyme, respectively. PEP residues are depicted in grey sticks, while ligands are represented in different coloured sticks. Red dotted lines indicate the presence of hydrogen bonds.

In case of compound **3**, it was observed that the compound binds slightly farther from the substrate binding site, with a binding energy of − 6.31 kcal/mol. The PEP residues surrounded by the compound include Arg252, Pro257, Tyr311, Leu338 and Leu354 (Fig. 4A). The nitrogen of each carbazole scaffold mediates hydrogen bonds with Arg252 and Leu338. Similarly, the aromatic rings were involved in the hydrophobic interactions. In case of compound **4**, it was observed that the compound binds closely to the substrate binding site and also interacted with a few residues in the substrate binding site, with a binding energy of − 7.73 kcal/mol. The PEP residues surrounded by the compound include Arg252, Cy255 (from S3 subsite), Asp256, Tyr473, Phe476 (from S1 subsite), Ile478, Arg643 (from S2 subsite), as well as Ser554 and His680 from catalytic site (Fig. 4B). The Arg463 observed to mediate hydrogen with one of the carbazole nitrogen while other residues were involved in π-stacking and hydrophobic interactions. Interestingly, in case of compound **5**, it was observed that the compounds bind within the substrate binding site with the binding energy of − 9.81 kcal/mol. The PEP residues surrounded by the compound include Phe173, Met235, and Cys255 from the S3 subsite, Arg643 from the S2 subsite, as well as Phe476 and Trp595 from the S1 subsite (Fig. 4C). Additionally, Ser554 and His680 from the catalytic triad, while other surrounding residues include Arg252 and Gly553. The arginine residues (252 and 643) mediate hydrogen bonds with the nitrogen of carbazole moiety, while other residues were involved in π-stacking and hydrophobic interactions. Similarly, in case of compound **6**, it was also observed that the compound also binds to the substrate binding site with the binding energy of − 10.04 kcal/mol. The PEP residues surrounded by the compound include Phe173 and Met235 from S3, and Phe476 from S1 subsite. In addition, Ser554 and His680 from the catalytic triad, while other surrounding residues include Val151, Arg252, Val578 and Tyr473. The carbonyl group in the compound mediated a hydrogen bond with Tyr473 while all the other residues were involved in π-stacking and hydrophobic interactions as depicted in Fig. 4D. In the case of compound **7**, it was observed that the compound binds very closely to the substrate binding site and exhibited the highest binding energy of − 10.66 kcal/mol compared to the other compounds. The PEP residues around the compound include Arg643 from S2 subsite while other surrounding residues include Arg252, Pro257, Gln208, Gly236, Gly237, Glu289 and Tyr473. As evident from Fig. 4E, the carbonyl group forms two hydrogen bonds with Arg252, and the nitrogen of the triazole ring mediates a hydrogen bond with Tyr473. Additionally, Gln208 facilitates a halogen bond with the compound. Furthermore, other residues participate in π-stacking and hydrophobic interactions, contributing to the stabilization of the protein–ligand complex. In the case of compound **8**, it was observed that the binding site for the compound is slightly distant from the substrate binding site, with an exhibited binding energy of -9.93 kcal/mol. The PEP residues surrounded by the compound comprise Gly237, Gly239, Ser250, Arg252, Pro257, Ser479, Ile480, Thr481, and Glu339. From the substrate binding site, only Cys255 from S3 subsite contributed in the compound's binding (Fig. 4F). The nitrogen of triazole ring mediate the hydrogen bond with Thr481 while other aromatic rings in the compounds were involved in the hydrophobic interactions with the surrounded residues. Finally, in case of compound **9**, the compound binds nearly to the S1 and S2 subsite with a binding energy of − 10.96 kcal/mol. The surrounded residues of PEP were included Gly237, Arg252, Phe476, Ile478, Val578, Arg643, val644 and His680 (Fig. 4G). The nitrogen of triazole ring and oxygen of methoxy group mediates three hydrogen bonds with Gly237 and Arg643. Similarly, the complex was further stabilized by a network of hydrophobic interactions mediated by aforementioned residues. The results molecular docking simulation inferred distinctive binding profile of each compound with the PEP enzyme, suggesting the potential inhibitory mechanisms.

### Molecular dynamics simulation

**Figure 5:**
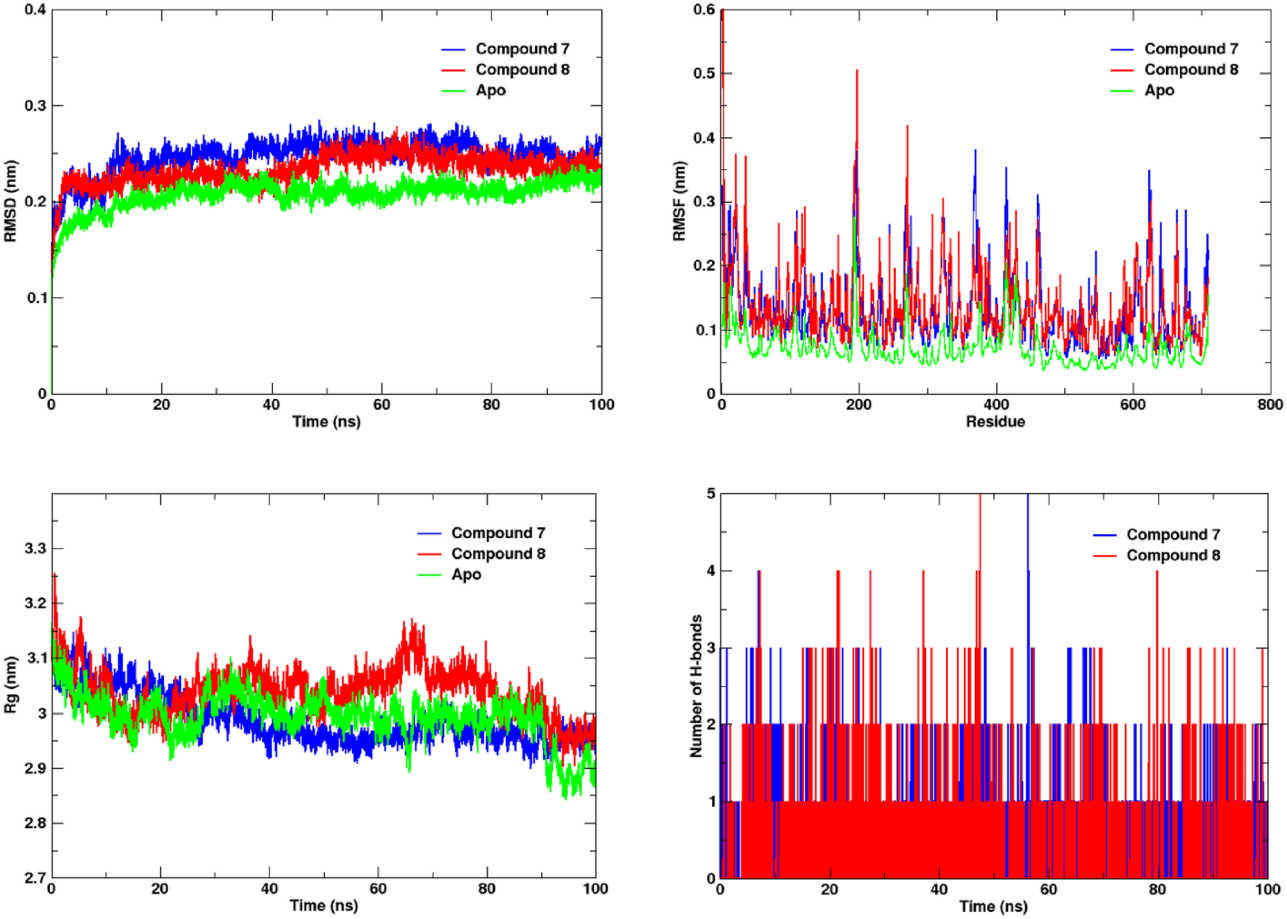
Analysis of molecular dynamics simulation results for the PEP enzyme and ligand complexes. Root Mean Square Deviation (RMSD) depicting the overall structural stability over time. Root Mean Square Fluctuation (RMSF) illustrating residue-level flexibility. Radius of gyration (Rg) reflecting changes in overall protein compactness. Hydrogen bond analysis highlighting the persistence of interactions throughout the simulation.

The molecular dynamics (MD) simulations of the PEP enzyme in complex with compounds **7**, **8**, and the apo enzyme were conducted over a period of 100 ns to assess the dynamic behaviour and stability of these systems. The root means square deviation (RMSD) analysis revealed that all three systems exhibited significant stability during the simulation and no major fluctuations were observed (Fig. 5). All the systems fluctuated between 0.15 and 0.27 nm while the average RMSD for the apo, compounds **7** and **8** complexes was 0.20 nm, 0.24 nm and 0.23 nm, respectively. Interestingly, a noticeable difference was observed in the RMSD plot of compounds **7** and **8** compared to the apo enzyme. The RMSD profiles of both compounds exhibited slightly higher fluctuations than those of the apo enzyme. These distinct dynamics profile may be attributed to the accommodation of both compounds into the region adjacent to the active site. The binding of the compounds may induce conformational changes, leading to enhanced flexibility in certain regions. Despite the higher RMSD of both compound complexes as compared to the apo system, it did not compromise the overall stability of the systems. The root mean square fluctuation (RMSF) analysis was performed to elucidate flexibility at the residue level for the apo enzyme, compounds **7** and **8** complexes. As expected, the RMSF of the apo system was lower as compare to the both compound complexes, with an average value of 0.07 nm, while both compounds exhibited a higher average RMSF of 0.13 nm (Fig. 5). This observed difference in flexibility could be attributed to the binding of compounds **7** and **8**, which might induce changes and change the dynamics of residues. However, it is pertinent to mention that all three systems exhibited fluctuations within the range of 0.05–0.4 nm, which is considered acceptable. The RMSF analysis revealed both intrinsic and ligand-induced flexibility within the PEP structure. Similarly, we also calculated the radius of gyration (Rg) to assess the structural compactness of the PEP enzyme in its apo form and in complex with compounds **7** and **8**. The analysis of Rg revealed interesting observations, the apo system projected the average Rg of 2.9 while for compound **7**, it remained at 2.9 nm as well nm. However, the complex with compound **8** exhibited a slightly higher Rg value of 3.0 nm, which can be attributed to the observed fluctuations, as depicted in Fig. 5. This observation suggests that the binding of compound **7** did not alter the compactness of the PEP enzyme, whereas the binding of compound **8** induced a conformational change that altered the compactness of PEP. During analysis of the trajectories of the compound **8** complex, an interesting observation was made: at around 62 ns, the loop region spanning from Asp159 to Leu168 underwent a transition into a beta sheet conformation. However, by 66 ns, this region reverted back to its original loop conformation and remained as such until the end of the simulation. This observation may be attributable to a peak observed in the Rg graph of the compound **8** complex (Supplementary Figure S6). Furthermore, the h-bond contacts were also investigated to evaluate the strength of interactions. Both compounds exhibited the ability to form four hydrogen bonds; however, the persistence of two hydrogen bonds during the simulation was noteworthy (Fig. 5). Taken together, the analysis of MD simulation results suggested significant stability in the protein–ligand complexes at the molecular level.

### Binding free energy

**Figure 6:**
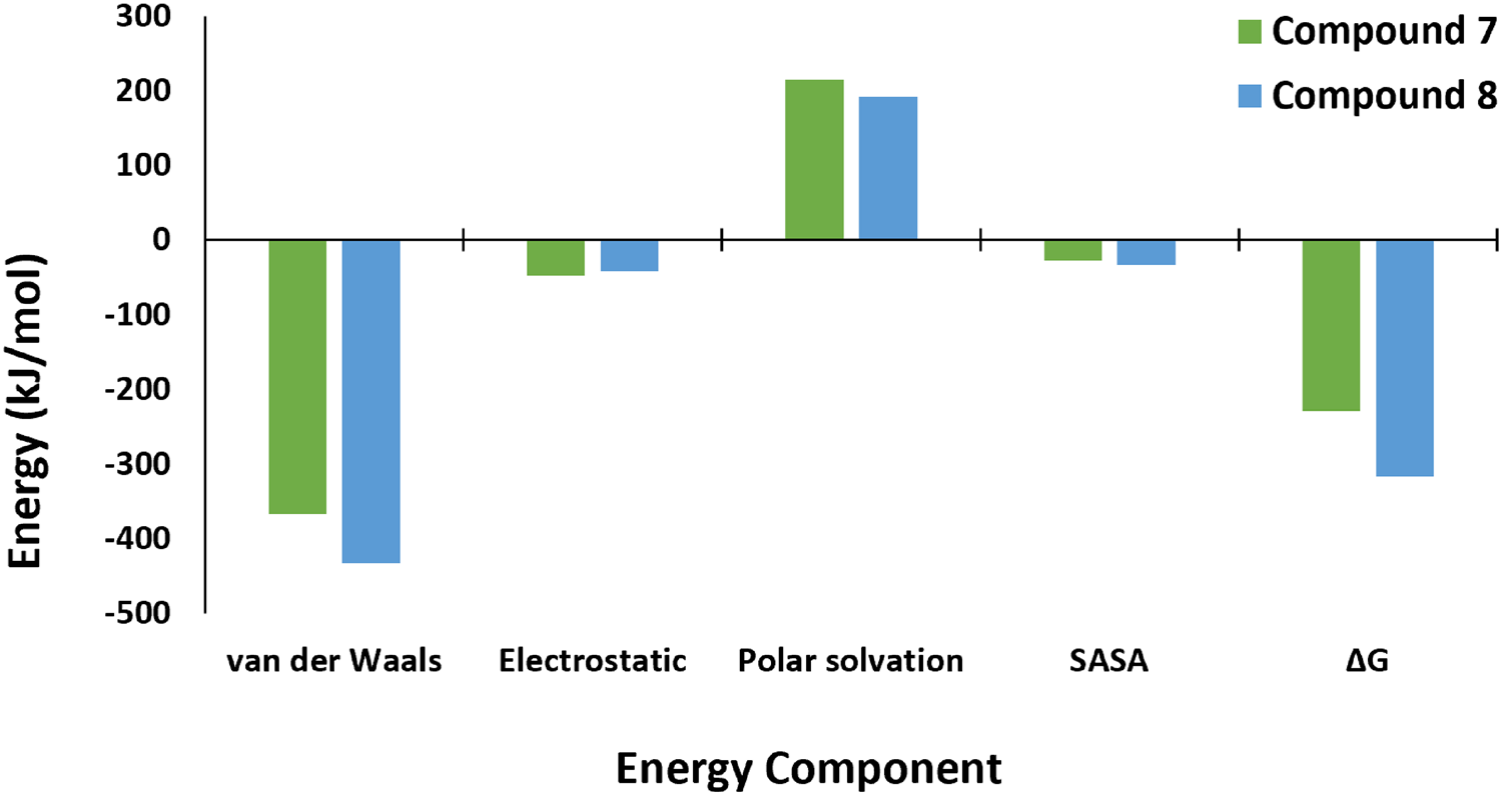
Binding free energy and individual energy components analysis using the MM/PBSA method for the PEP enzyme-ligand complexes.

The relative binding free energy using the MM/PBSA method of compounds **7** and **8** complexes with the PEP enzyme was computed to elucidate the thermodynamic stability and energetic contributions governing the binding interactions. The calculated binding free energy (ΔG) for compound **7** complex was found to be − 228.05 kJ/mol, while for compound **8** complex, it was more favourable at − 316.18 kJ/mol, indicating a stronger binding affinity for compound **8** in comparison of compound **7** (Fig. 6). Further the analysis of individual energy components revealed interesting details (Fig. 6). Compound **8** complex displayed higher van der Waals interactions, which suggested a potential contribution from nonpolar forces in stabilizing the complex. However, compound **7** complex exhibited the higher electrostatic interactions, highlighting the role of charged interactions in complex stability. The polar solvation energy was positive for both compound complexes and relatively higher for compound **7** (214.22 kJ/mol) compared to compound **8** (192.48 kJ/mol). Additionally, the solvent-accessible surface area (SASA) energy showed less contribution for both the complex, with the SASA values of − 28.11 kJ/mol and − 33.93 kJ/mol for compounds **7** and **8**, respectively.

### ADMET analysis

In silico prediction of ADMET (Absorption, Distribution, Metabolism, Excretion, and Toxicity) profiles during the early stages of drug development is crucial for reducing late-stage failures and streamlining the selection of promising drug candidates and lead optimization. Herein, the ADMET profile of compounds **3–9** were evaluated by AdmetSAR webserver. Interestingly, all compounds were predicted to penetrate the blood–brain barrier, which suggested a potential central nervous system activity of the compounds. Similarly, none of the compounds were predicted to be carcinogenic. All compounds, with the exception of compound **3**, were predicted to exhibit hepatotoxicity, respiratory toxicity, and mitochondrial toxicity. Compound **3**, on the other hand, was not predicted to demonstrate mitochondrial or respiratory toxicity. Moreover, all compounds exhibited no nephrotoxicity, except for compound **7**. Similarly, all compounds displayed good oral bioavailability and high gastrointestinal absorption, which suggested their potential suitability for efficient absorption and systemic availability in the body.

## Methodology

### General procedure of bicarbazole-linked triazoles synthesis

Acetophenone azide (5a–f) was added to 0.5 mmol of bicarbazole acetylene (4) in a DMF: water combination (3: 2 v/v) at room temperature. After that, 0.7 mmol of sodium L-ascorbate (0.7 mmol) and 0.5 mmol of copper (II) sulphate pentahydrate (CuSO4.5H2O) were added and mixed for two hours. Ice cold water was added to the mixture when it had been finished, and it was left for 2 h until precipitates had formed. To get pure products in yields of 82–87%, precipitates were filtered, washed with 25% ethyl acetate in n-hexanes, and purified using column chromatography (eluent: 20–90% ethyl acetate in n-hexanes).

### In vitro inhibitory assay

Prolyl endopeptidase inhibitory activity was measured using a slightly modified version of Yoshimoto et al. spectrophotometric technique. In a 96-well plate, 140 µL of sodium phosphate buffer (50 mM, pH 7.0), 20 µL of the test substance (0.5 mM in methanol), and 20 µL of prolyl endopeptidase solution (0.02 U/well) were added to provide a total reaction volume of 200 µL. Blank contained 20 µL methanol, instead of the test compound, while Z-prolyl-prolinal (0.5 mM) was used as the positive control. After the reaction mixture had been incubated at 30 °C for 10 min. Now 20 µL of the substrate solution, Z-Gly-Pro-4-nitroanilide (0.4 mM in 1,4-dioxane), was added, and a 96-well plate reader was used to continually monitor the change in absorbance at 410 nm for 30 min (SpectraMax-384, Molecular Devices, CA, USA). The reaction mixture's ultimate methanol and 1,4-dioxane concentrations were 10% v/v. In 96-well microplates, each reaction was carried out in triplicate.

### Statistical analysis

The current study's experiments were all carried out in triplicate, and the data were presented as the standard error of the mean (SEM). Using the statistical software EZ-FIT enzyme kinetics, the IC_50_ values were determined (Perrella Scientific, Inc., Amherst, NH 03031, USA). To determine the % inhibition of compounds, the Excel formula below was developed showed in Eqs. (1) and (2).

1 $$\begin{array}{*{20}c} {\% Inhibition = 100 - \left( {\frac{{O.D_{test compound} }}{{O.D_{control} }}} \right) \times 100 } \\ \end{array}$$

2 $$\begin{array}{*{20}c} {SE = \frac{\sigma }{\sqrt n } } \\ \end{array}$$

### Kinetic studies

In kinetic study PEP (0.02 U/200 L) was incubated with a range of inhibitor different concentrations for 10 min at 30 °C. The substrate was often utilized at doses between 0.2 and 0.5 mM. The substrate solutions (Z-Gly-Pro-pNA) were added at four different concentrations (0.2, 0.3, 0.4, and 0.5 mM), starting with the lowest concentration and working up to the highest concentration, to begin the catalytic reaction. Using a 96-well plate reader, the catalytic activity of PEP was measured at 410 nm (SpectraMax-384, Molecular Devices, CA, USA).

### Cytotoxicity evaluation of the tested compounds

To investigate the cytotoxic effect of these compounds we proceeded all the active compounds 3–9 for their *in*-*vitro* study by using BJ cell line. Here we designed our experiment with a specific count of the cells with 6 × 104 cells/well were employed into the plate. In each well we added 100 µL followed by 24 h incubation at specific temperature 37 °C in 5% CO_2_. After that, the 96-well plate was incubated with 50 µL/well (60 µM) of the tested compounds along with 150 µL/well fresh media for 48 h at the similar specific experimental conditions. After 2nd time of incubation, the old media was replaced with 200 µL yellow MTT dye in each well according to 1:10 ratio with the media followed by 4-h incubation. In the next step the dye was removed completely and DMSO (Dimethyl sulfoxide) cell culture grade 100 µL/well was employed into the 96-well plate to dissolve the produced formazan crystals of MTT dye converted into purple. At the end of experiment the obtained changes in the (OD) optical density was monitored at 540 by using spectrophotometer.

### Molecular docking study

Molecular docking simulation is the most efficient computational technique to determine the binding mode of a small molecule into the binding site of target protein of interest. Herein, docking studies were performed to determine the mechanism of observed anti-prolyl endo peptidase activity. The crystal structure of PEP was obtained from Protein DataBank repository (PDB ID 1QFS)^35^. The structure was then subjected to preparation using the Structure Preparation module of Molecular Operating Environment (MOE) v.2022.02 software (https://www.chemcomp.com/) followed by protonation^37^. Afterwards, the minimization was carried out using the Amber10: EHT force filed. As the compounds demonstrated mixed-type inhibition, indicating their binding occurs at a site distinct from the substrate binding site, we performed two docking approaches. Firstly, blind docking was employed, enabling the compounds to dock freely to the target protein without predefined constraints. Subsequently, a second docking method utilized the coordinates of the bound substrate (Z-Pro-Prolinal, ZPP), defining the docking grid based on the substrate's coordinates. The analysis of compounds binding energy (low docking score) led to the identification of a binding target site. The docking paraments were set as same as followed by Khan et al., used for the docking of PEP inhibitors^38^. In brief, the Triangular Matcher was employed as the placement method, complemented by London dG and WSA/GBVI dG for scoring and rescoring functions, respectively. Ten conformations were generated for each compound, and the top-ranked conformations were visually examined. All the graphics were rendered by Chimera v.1.17.1 software (https://www.cgl.ucsf.edu/chimera/)^39^.

### Molecular dynamics simulation

In order to determine the time dependent stability of the protein–ligand complexes, MD simulation was carried out using GROMACS (GROningen MAChine for Chemical Simuation) v.2022 (https://www.gromacs.org/)^40^. In total, three systems were simulated including apo PEP protein and in complex with compound 7 and 8. The ligands were prepared by using Automated Topology Builder v.3.0 (https://atb.uq.edu.au/) while the parameters of the PEP protein were generated by GROMOS96 54a7 force field. All three systems were placed in a cubic box containing the TIP3P water model, each positioned at a distance of 1.0 nm from the edges of box. The counter ions were added to neutralize the systems and further subjected to minimization. The steepest descent algorithm was employed during the minimization process. Subsequently, the equilibration of 200 ps was performed in the NVT and NPT ensemble using the isobaric-isothermal algorithm. The long-range electrostatic interactions were treated by Particle Mesh Ewald algorithm while LINCS algorithm was used to constrained the h-bonds. The final production of 100 ns was carried out at a time step of 2.0 fs. The simulated trajectories were analyzed by plotting key parameters, including root mean square deviation (RMSD), root mean square fluctuation (RMSF), radius of gyration (Rg), and hydrogen bonds, over the course of the 100 ns simulation.

### Binding free energy

Herein, relative binding energy of PEP enzyme in complex with compounds **7** and **8** was computed using the by MM/PBSA (Molecular Mechanics Poisson–Boltzmann Surface Area). This analysis was based on the trajectory snapshots extracted from the most stable part of simulation trajectories using the g_mmpbsa script provided by Kumari et al.^41^.

### ADMET analysis

In silico prediction of ADMET (Absorption, Distribution, Metabolism, Excretion, and Toxicity) profiles of compounds 3–9, were predicted from AdmetSAR v.2.0 webserver available at http://lmmd.ecust.edu.cn/admetsar1/. For the analysis, the SMILE notation of each compound was submitted to the webserver^42^.

## Conclusion

Because excessive PEP enzyme overexpression in the brain causes neurodegenerative conditions like dementia, Alzheimer's, and Parkinson's disorders. There is currently no FDA-approved medication that can inhibit the PEP enzyme's hyperactivity. Keeping in view of this fact, the current work set out to identify novel PEP inhibitors that might be used as leads for the development of new drugs to treat prolyl endopeptidase-related disorders. Hence, the development of new inhibitors is an immense need to overcome on neurodegenerative disorders. In this context we identified seven new inhibitors with high potency ranging from 26 to 63 µM. Furthermore, kinetic study of compounds **7** and **8** was performed resulted into mixed type inhibition. Furthermore, molecular docking studies with MD simulation and binding free energy revealed that the compounds bind in close proximity to the substrate binding site with significant affinity and stability, aligning with the experimental results. These significant findings insight into the potential therapeutic approach for the treatment of neurodegenerative disorders and may serve as potential leads for the identification of new drug candidates for to tackle neurodegenerative disorders.

### Supplementary Information

Supplementary Figures.

## Data Availability

The datasets used and/or analysed during the current study available from the corresponding author on reasonable request.
