# Supplementary material for: Exploring bi-carbazole-linked triazoles as inhibitors of prolyl endo peptidase via integrated in vitro and in silico study
Source: Sci Rep. 2024 Apr 1;14:7675. doi: 10.1038/s41598-024-58428-6 (PMC10985113; doi:10.1038/s41598-024-58428-6)
Supplement: Supplementary file 1 — Supplementary Figures. [file 41598_2024_58428_MOESM1_ESM.docx]

**Exploring Bi-carbazole-linked Triazoles as Inhibitors of Prolyl Endo Peptidase *via* Integrated *In Vitro* and *In Silico* Study**

**Saeed Ullah ^aϮ^, Farheen Mansoor ^bϮ^, Salman Ali Khan ^c^*, Uzma Jabeen ^d^, Amany I. Almars ^e^, Hailah M. Almohaimeed ^f^, Ahmed M. Basri ^e^, and Fahad M. Alshabrmi ^g^**

^a^ Natural and Medical Sciences Research Center, University of Nizwa, Birkat-ul-Mouz 616, Nizwa, Sultanate of Oman

^b^ Dr. Panjwani Center for Molecular Medicine and Drug Research, International Center for Chemical and Biological Sciences, University of Karachi, Karachi-75270, Pakistan.

^C^ Tunneling Group, Biotechnology Centre, Doctoral School, Akademicka 2, Silesian University of Technology, 44-100 Gliwice, Poland

^d^ Department of Biochemistry, Federal Urdu University of Karachi, Gulshan-e-Iqbal, Karachi – 75300, Pakistan

^e^ Department of Medial Laboratory Sciences, Faculty of Applied Medical Science, King Abdulaziz University, Jeddah 21589, Saudi Arabia

^f^ Department of Basic Science, College of Medicine, Princess Nourah bint Abdulrahman University, P.O.Box 84428, Riyadh 11671, Saudi Arabia

^g^ Department of Medical Laboratories, College of Applied Medical Sciences, Qassim University, Buraydah 51452, Saudi Arabia

Saeed Ullah ahmedsaeedkhan872@gmail.com

Farheen Mansoor farheenmansoor03@gmail.com

Uzma Jabeen^d^ [dr.uzmajabeen@fuuast.edu.pk](mailto:dr.uzmajabeen@fuuast.edu.pk)

Salman Ali Khan salman.ali@polsl.pl.

Amany I. Almars aialmars@kau.edu.sa

Hailah M. Almohaimeed [hmalmohaimeed@pnu.edu.sa](mailto:hmalmohaimeed@pnu.edu.sa)

Ahmed M. Basri [abasri@kau.edu.sa](mailto:abasri@kau.edu.sa)

Fahad M. Alshabrmi fshbrmy@qu.edu.sa

*** Correspondence:**

**Salman Ali Khan**

[salman.ali@polsl.pl](mailto:salman.ali@polsl.pl)

**^Ϯ^**These authors have contributed equally.


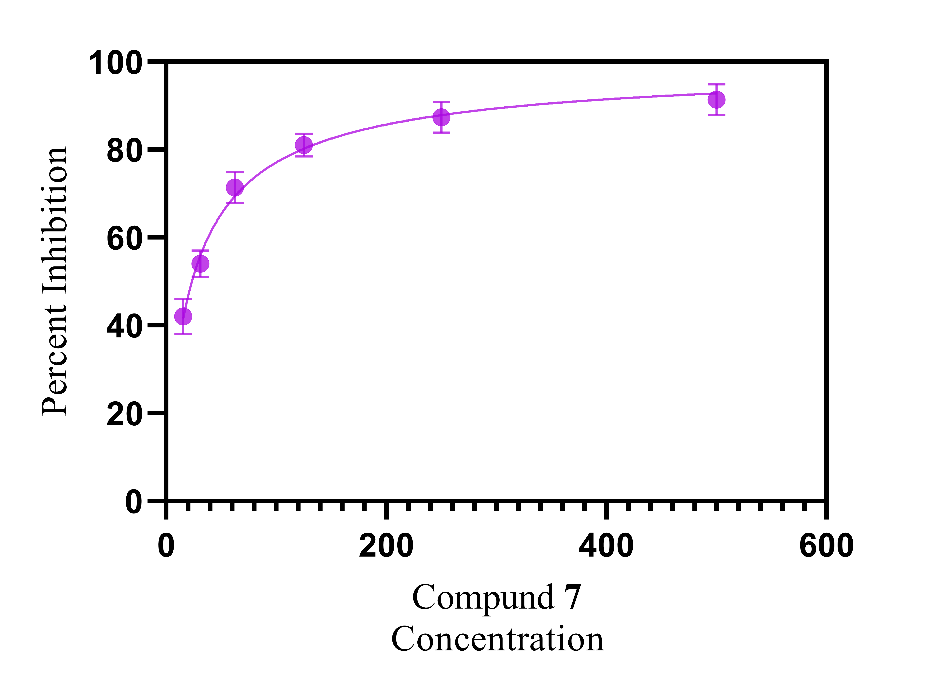


**Figure** S**1**. Dose curve response against prolyl endo peptidase for compound **7**.


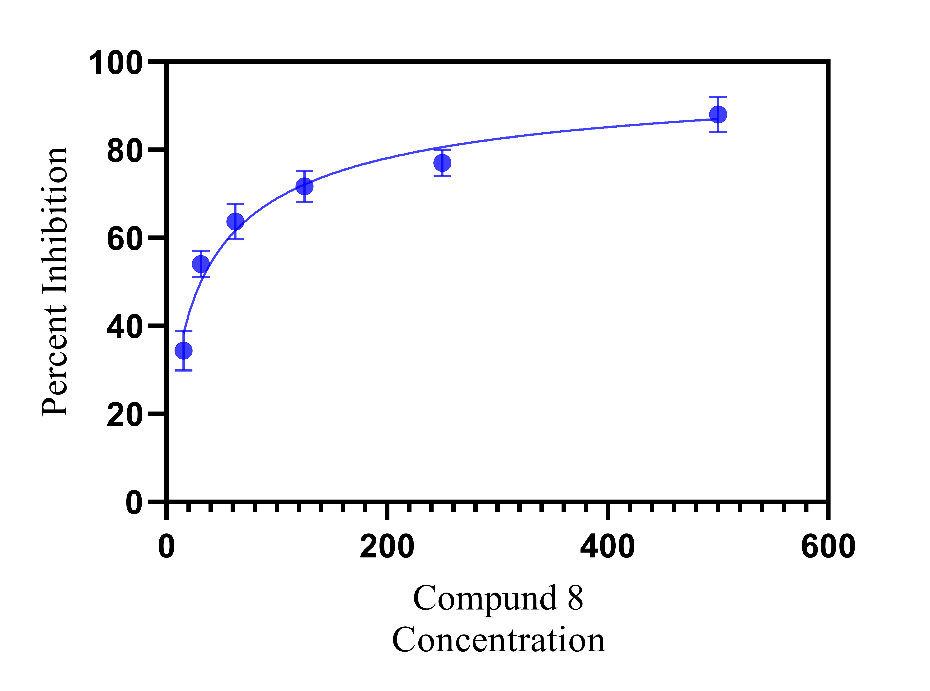


**Figure** S**2**. Dose curve response against prolyl endo peptidase for compound **8**.

**Figure S3.** The cytotoxicity effect of compounds **3**-**5** on BJ cell line.

**Figure S4.** The cytotoxicity effect of compounds **6**-**8** on BJ cell line.

**Figure S5**. The cytotoxicity effect of compounds **8** and **9** on BJ cell line


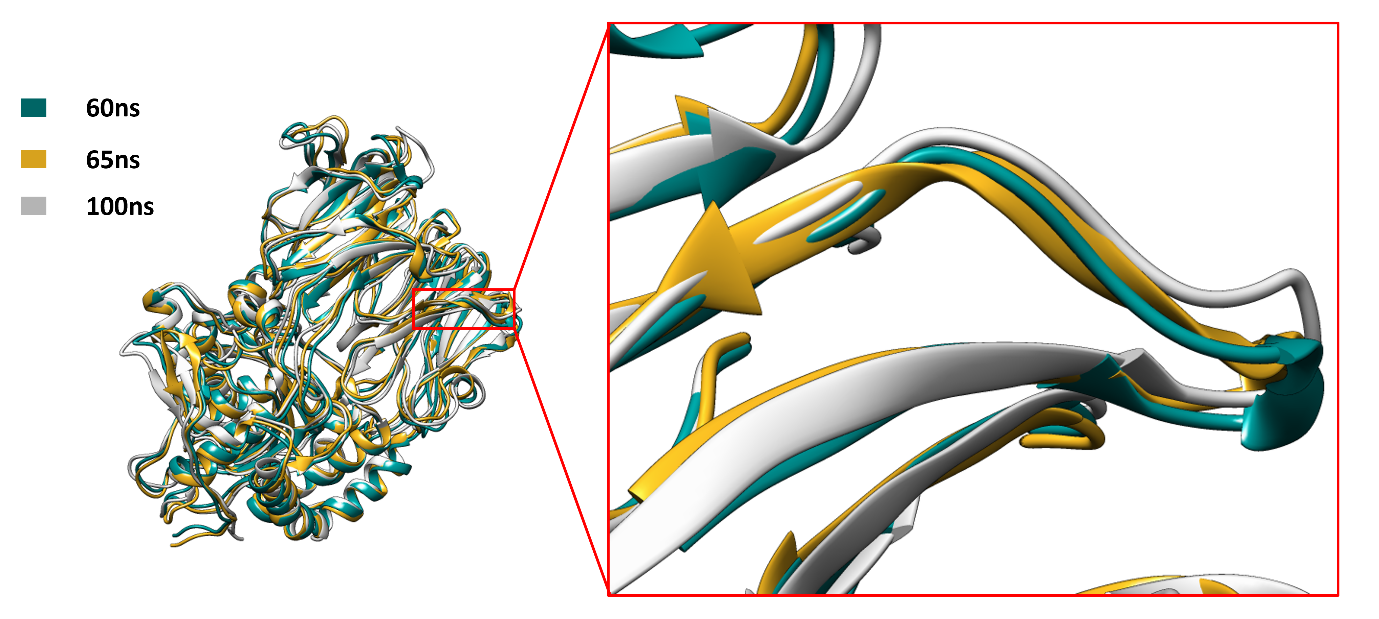


**Figure S6**. Structural alignment of frames at 60 ns (dark cyan), 65 ns (goldenrod), and 100 ns (light grey) of compound **8** complex, revealed dynamic changes in the loop region from Asp159 to Leu168. The structure at 60 ns and 100 ns exhibited a loop conformation, while at 65 ns, a transition to a beta sheet was observed in this region.
